# Systematic Review of Phytotherapeutic Treatments for Different Farm Animals Under European Conditions

**DOI:** 10.3389/fvets.2018.00140

**Published:** 2018-06-22

**Authors:** Lena-Mari Tamminen, Ulf Emanuelson, Isabel Blanco-Penedo

**Affiliations:** ^1^Veterinary Epidemiology, Department of Clinical Sciences, Swedish University of Agricultural Sciences, Uppsala, Sweden; ^2^Animal Welfare Subprogram, Institute of Agrifood Research and Technology, Girona, Spain

**Keywords:** PICOS approach, veterinary phytotherapy, botanical treatment, medicinal plants, efficacy evaluation, antibiotic resistance, effectiveness

## Abstract

The emergence and spread of antibiotic resistance is a growing global threat and has aroused a global interest in limiting antibiotic use in animal agriculture. As we are entering the post-antibiotic era, there is a growing need for identifying alternatives to antibiotics for prevention and treatment. Although phytotherapeutic remedies are available and are used in Europe, evidence of their efficacy is currently very limited but is nevertheless still necessary. A systematic review of phytotherapy used for the treatment and prevention of infectious diseases in farm animals has been conducted using the PICOS approach. Analysis and extraction of information from studies was performed according to a protocol, and included: publication year and source, research body, species, farming system, purpose of application, disease in focus, diagnostic method, remedy used (origin and ingredients of the remedy), producer of remedy, way of administration, study design, and control groups, measure of effect, and outcome of the study. A total of 1,705 papers were screened, but only 44 (comprising 53 independent studies) met the inclusion criteria. The majority of the scientific studies identified show limitations in the study design as well as in presentation and standardization of the botanical remedies studied, which limits the possibilities of drawing firm conclusions. Preventive effect was investigated in 89% of the studies. Half of the studies reported uncertain effects of the botanical remedies on subclinical measures in comparison to a control group. The review highlights unresolved questions such as the need for standardized controlled trials and for improved standardization of the botanical products by analytical methods, such as high-performance liquid chromatography. It is necessary to consider this in order to achieve replicable outcomes and establish efficacy and safety of phytotherapy in farm practice. The manageability of alternative medicine in farm animals also warrants further investigation so as to ascertain whether all necessary preconditions for their use are being granted.

## Introduction

Since their advent in the last century, antibiotics have become essential for the treatment of infectious diseases in both humans and animals and have enormously improved the health of the population as well as food security and safety. However, studies have demonstrated that routine use of antibiotics may lead to antimicrobial resistance, and the rapid development of resistance has emerged as a major global public and animal health issue (1–3). Both antimicrobial resistance and a possible future limitation on antibiotic use for animal agriculture could have an impact on global food security and safety as well as on animal health and welfare (4). As we are entering the post-antibiotic era, there is a growing need to identify alternatives to antibiotics.

The antimicrobial activity of plant oils and extracts has been recognized for many years (5). Over the last two decades, the study of phytotherapy—modern scientific herbal medicine in which the molecular interactions between plant components and cells are studied to provide evidence-based treatments—has gained strength in the scientific community (6–9). Despite its promising potential for replacing antibiotic treatments (10) research and reviews on the use of botanical products in animal agriculture have mainly focused on alternatives to antibiotic growth promoters or boosters of general health (11–13). However, where the essence of treatment such as curative, metaphylaxis, and prevention, the three different circumstances for antimicrobial treatment, might has not been well formulated and recognized in the case of alternative treatments (14). On the market, the availability of veterinary herbal medicinal products varies greatly among EU countries (15), and feed additives available to farmers are heterogeneously used to improve performance and general health with the main purpose of prevention (16).

The aim of this study was to evaluate the current potential of phytotherapeutic products for replacing antibiotics for treatment and prevention (since prevention reduces the need to treat) of specific diseases of cattle, poultry, and swine in Europe. This was done by performing a systematic literature review according to PRISMA guidelines of studies using phytotherapeutic remedies and evaluating their study design and treatment efficacy.

## Materials and methods

This review is part of a European Project (No. 311824) called IMPRO (http://www.impro-dairy.eu/). The project aimed to overcome weak points in current health management strategies on organic dairy farms and addressed, for instance, the assessment of the manageability of alternative treatments according to the state-of-the-art for different farm animals.

### Literature search

The literature search was performed between 12th and 14th of February 2014 in the databases Web of Science, Pubmed, and Scopus according to PRISMA guidelines (Preferred Reporting Items for Systematic Reviews and Meta-Analyses) (17). PRISMA guidelines provide an evidence-based minimum set of items regarding the methodology and identification of publications as well as reporting in these types of reviews. The search terms were defined in collaboration with a professional librarian specialized in scientific databases using the PICOS approach (population, intervention, comparison, outcome, and study design), and exclusion of terms was added to remove a large number (>10,000) of off-topic publications. The final search terms were:

bovine OR cattle OR cow OR cows OR heifer OR heifers OR (dairy AND calf) OR (dairy AND calve) OR (dairy AND calves) OR calve OR calves OR calf OR calfs OR (dairy AND herd) OR (dairy AND herds) OR (dairy AND farm) OR (dairy AND farms) OR swine OR pig OR pigs OR piglet OR piglets OR weanlings OR weanling OR weaner OR weaners OR fatteners OR fattener OR porcine OR poultry OR hen OR hens OR laying hen OR chicken OR chickens

AND

*((herbal OR herb) AND (medicine OR medicinal OR extract OR treatment OR product OR tincture OR drug OR remedy OR remedies OR supplement OR preparation)) OR phytotherapy OR phytotherapeutic OR phytoceutic OR phytochemical OR phytogenic OR phototherapeutic OR medicinal plant OR plant extract OR ethnoveterinary medicine OR (bioactive AND (plant OR herb OR drug)) OR essential oil OR organic acid OR genistein OR capsaicin OR carotenoid OR saponin OR saponins OR tannin OR tannins OR polyphenol OR polyphenols OR lignan OR lignins OR aromatic acid OR glucosinolate OR glucosinolates OR thymol OR (natural AND (terpene OR terpenes OR flavonoid OR flavonoids OR flavone))*.

AND

comparison OR compared OR prevention OR preventive OR treatment OR treatments OR treating OR ((control OR outbreak) AND (disease OR infection OR diseases OR infections))

AND

(*efficacy OR effectiveness OR effect OR efficiency OR capability OR potency OR success OR ineffectiveness OR ineffective OR failure OR inefficiency)*

NOT

(vaccine OR vaccination OR “in vitro” OR mice OR rat OR “guinea pig” OR “shelf life” OR filets OR “ground meat”)

**Figure 1:**
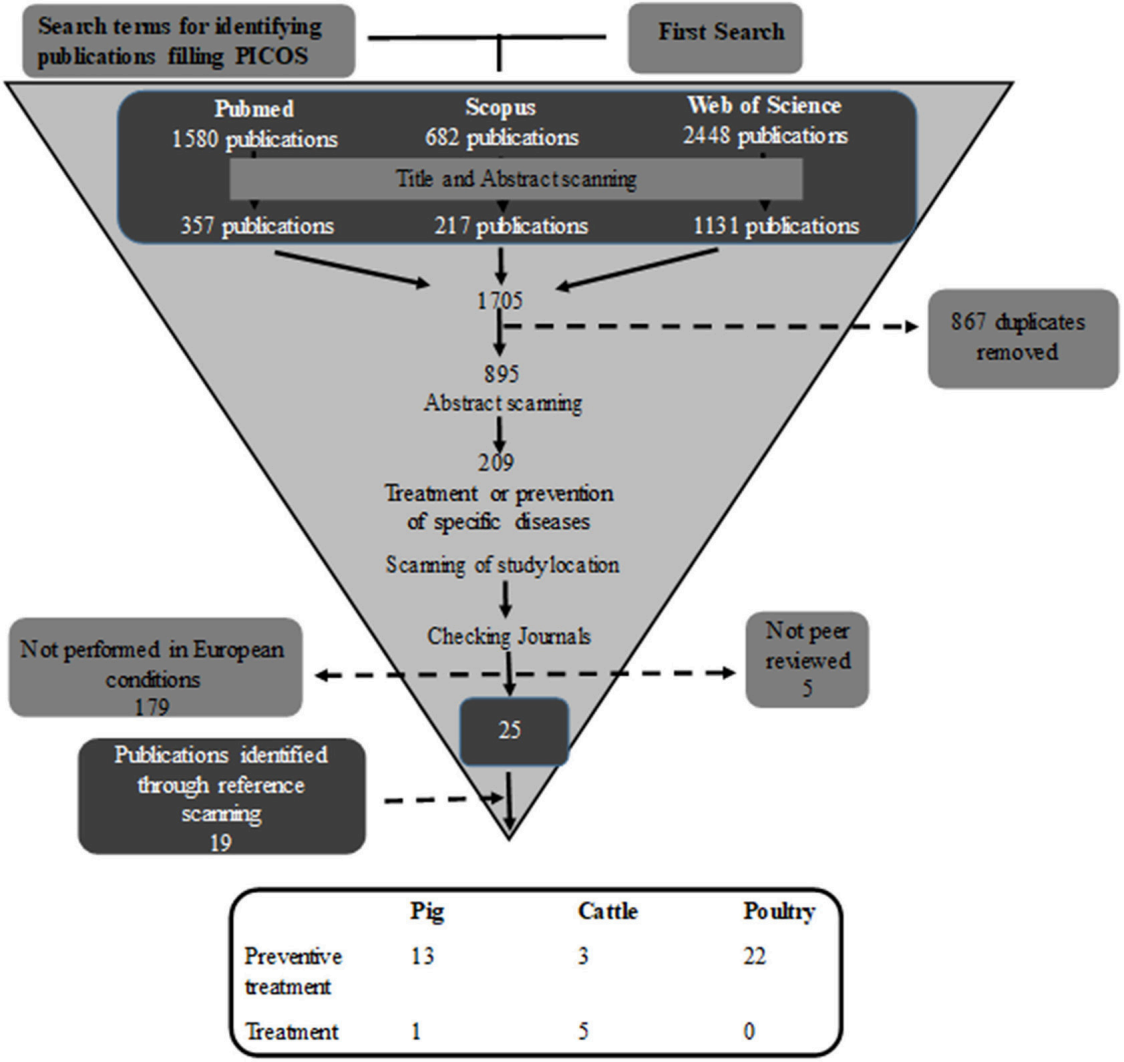
Search process for identifying publications where phytotherapeutic remedies are used to treat/prevent disease.

The period of publications considered was from year 2000 until the starting time of the search. The search and the filtering process (Figure 1) were performed by the same person in order to achieve consistency.

#### Inclusion and exclusion criteria

Studies in peer-reviewed scientific journals performed under European conditions (with respect to housing, breed, and intensive farming) where the effect of one or several substances derived from plants were used as treatment or to prevent a specific infection, production disease or symptom of disease on cattle, poultry, or pigs. Studies where phytotherapeutic substances were given with the aim of reducing shedding of pathogens were included, as this use could have a preventive effect on herd level.

Studies performed in conditions not comparable to European animal husbandry with respect to breeds, housing, and farming system were excluded as were studies published in journals that did not supply information of a peer review process where excluded from the review. In addition, studies of the treatment effect on general or physiological parameters not associated with a specific disease or specified clinical symptoms, such as growth, general mortality by unspecified reason and changes in microbiota or immune system were excluded from the review.

## Identification of literature for review

Publications that included more than one experimental study were extracted as separate studies for the evaluation and comparison of data. Analysis and extraction of information from studies was performed according to a protocol and included: publication year and source, research body, species, location/farming system, purpose of application, disease in focus, diagnostic method, remedy used (origin and ingredients of the remedy), producer of remedy, route of administration, study design, and control groups, measure of effect, and outcome of the study. A gold standard for presenting phytotherapeutic remedies was acquired from “Authentication and quality assessment of botanicals and botanical products used in clinical research” in Evaluation of Herbal Medicinal Products in 2009. The information extracted from eligible studies is fully presented in Supplementary Material 1 (study design and outcomes) and Supplementary Material 2 (phytotherapeutic treatments).

The use of the phytotherapeutic substance was classified as a treatment or prophylactic. When substances were used on animals with established disease, meaning clinical symptoms, or other pathological changes detected by analytical diagnostic methods, they were considered to be used as treatment. The use was classified as prophylactic when the substance was given to healthy animals before infection/disease contraction so as to prevent or reduce the effect of a specific pathogen/pathogens or reduce the incidence of disease in a population. Studies where experimental infection or other disease inductions were performed on the same day as the phytotherapeutic remedy was started were categorized as prophylactic studies because infection or disease was not established on the day the treatment began.

According to the efficacy of the botanical remedies, studies were sorted into four categories. Category A included remedies that significantly reduced clinical signs (including body temperature) and mortality. In category B were placed the substances that caused significant positive effects on subclinical measures in comparison to a control group. If the substance gave small, not statistically significant differences or significant differences that were not clearly associated with the disease compared to control groups, the result was classified as uncertain (category C). If no effect at all was reported, the study was categorized as D.

## Results

The literature search gave a large number of publications (*n* = 1,705), but throughout the review process most were excluded as they did not fulfil the inclusion criteria (Figure 1). Of the remaining 44 publications, 22 were performed on poultry, 14 on pigs, and eight on cattle. In total, these 44 publications included 53 separate studies. The majority of the studies (89%) investigated the preventive effect of phytotherapeutic products against specific diseases, while only six studies, five in cattle, and one in pigs, investigated the effect of treating an established disease after diagnosis. Of the 53 studies 44 studies were performed in a controlled experimental environment and nine were performed in a farm conditions. Of the studies performed on farms one studied poultry, four cattle, and four pigs.

**Figure 2:**
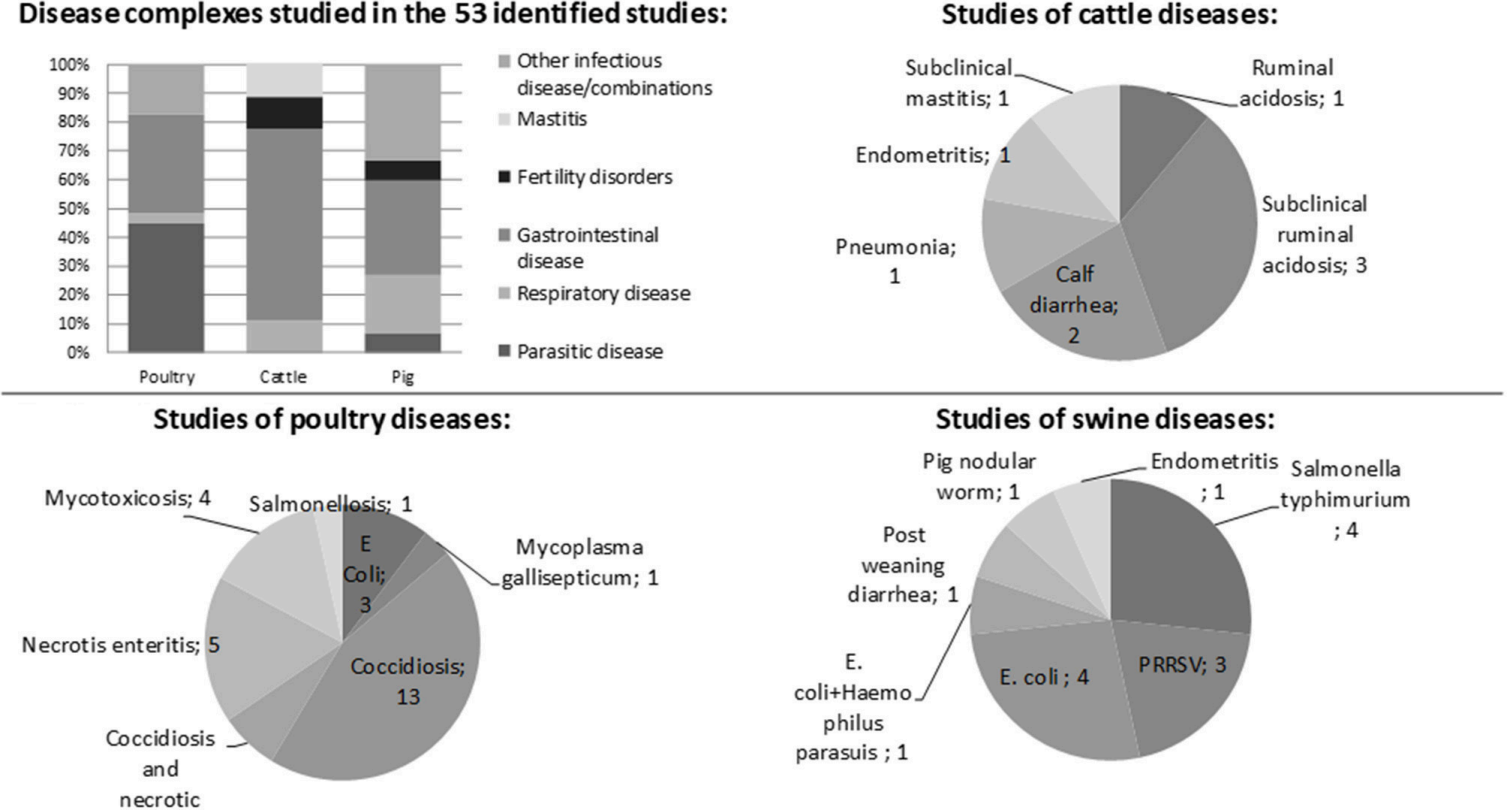
Diseases studied in the 53 identified studies.

The diseases studied in the publications are presented in Figure 2. They were dominated by infectious diseases of a specific pathogen (*n* = 44) or symptoms commonly caused by infectious origin (*n* = 5). Four studies, all of subclinical or clinical ruminal acidosis, were not associated with infectious disease.

### Study design

**Figure 3:**
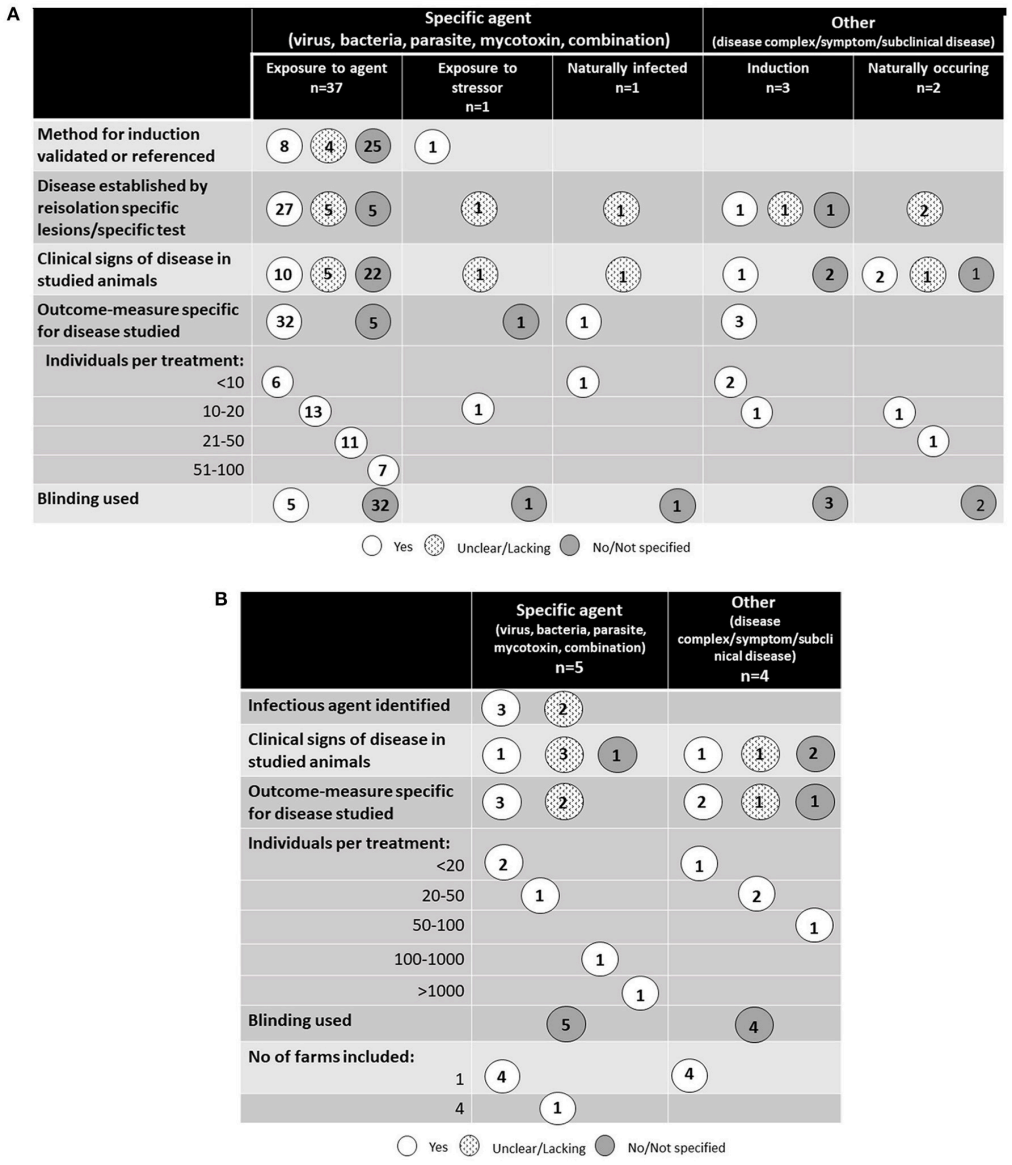
**(A)** Study design of the 44 studies identified in the literature review that were performed on controlled experimental conditions. **(B)** Study design of the nine studies identified in the literature review that were performed on farm conditions.

A schematic overview of the study designs for the 44 studies performed in controlled experimental conditions are presented in Figure 3A and the nine studies performed in farm environment are presented in Figure 3B. All studies except one were randomized trials in which animals were randomly assigned to different treatment and control groups. One study of ruminal acidosis was a cross-over design with eight individuals and a 15-day long transition period between treatments (18). Blinding was only reported in five of the 53 identified studies (19) (one study); (20) (four studies). In these specific surveys, the blinding was done on a laboratory level where the person interpreting pathological and histological changes did not know which group the individual animals came from. The number of animals included in the studies varied, but half of the studies used fewer than 20 animals per treatment group. Two studies, both performed in a commercial farm environment (21, 22) included more than 100 animals per treatment group. No power calculations regarding the number of treated animals were presented in any of the studies.

#### Experimental induction of disease

The most common induction of disease was experimental infection with a pathogenic organism or toxin derived from a pathogenic organism (37 studies). For poultry, this included all studies except one, and for pigs 10 out of 14 studies, respectively. Induction was not used in any of the five studies of disorders associated with infectious origin in cattle. Experimental induction was only used in one study of pneumonia (23), but in this study induction was not performed by infection with a pathogen; symptoms of pneumonia were instead induced by administering mineral oil to the lung through a tracheal catheter. In the remaining studies, animals were infected naturally from their environment.

The severity of disease and how the presence of disease was measured varied between studies. Twenty-eight studies reported no clinical signs, i.e., subclinical disease, and 11 studies reported a low frequency of clinical signs indicating low level of disease. In the 39 studies using experimental induction, 11 reported the use of a validated or a previously validated dose, although in three of these no reference to the original study was provided. For the remaining studies, re-isolation of the infectious agent and/or specific histological or pathological changes were common indicators of successful infection. However, in four of the studies only unspecific measures, not related to the disease, were used as both indicator of the disease and measure of outcome. Of the 10 studies where spontaneous disease was studied, five established disease by isolation of an infectious agent or disease specific changes. In the remaining studies, clinical symptoms or indirect measures were studied.

In general, the studies did not specify who performed the diagnostic procedure except in one of the studies on dairy calves by Oliveira et al. (24), where the research team performed the diagnostic procedure. No information on whether the research team contained veterinarians or other professions was given.

Large varieties of outcome-measures were studied. Some studies included several outcomes directly and/or indirectly linked to the disease while others reported only a few specific outcome measures. The majority of the studies (*n* = 46) included unspecific measurements indirectly associated with the disorder, such as, for example, production parameters (body weight, feed intake, and average daily gain) and mortality, in general in combination with more specific outcomes associated with the disease. Specific measures, such as rate of re-isolation of the pathogen or specific histological, pathological, and clinical changes for the disease in each individual treated, were used in 41 of the 53 studies. Nine of the remaining 12 studies did not use specific measures and included animals with no or very low levels of clinical symptoms, making the studies rely heavily on indirect tests. The remaining three studies, all studying the effect on *Escherichia coli*-induced diarrhea in calves or pigs, included animals with clinical signs. In animals from those studies, *E. coli* was isolated, but it remained unclear whether *E. coli* was the only causative agent of the diarrhea.

## Control groups

### On farm studies

For the studies performed in farm conditions, the most common control group was a non-treated control group which was included in seven out of nine studies. In three of these an additional control group receiving an alternative treatment was included. Two studies did not use any negative control group (given no treatment or placebo) but compared the effect of the botanical remedy with a control group receiving an alternative therapy type. One study compared the phytotherapeutic treatment with an injection of antibiotics on diarrhea in dairy calves (25) and one compared the effect of the phytotherapeutic treatment on endometritis cattle with one group receiving local application of Lotagen and one group receiving hormonal treatment with prostaglandins (26).

#### Controlled environment

**Figure 4:**
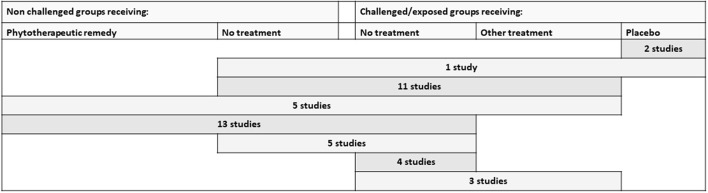
Different types of control groups and the combinations included in the identified studies performed under controlled experimental conditions. Other treatment includes allopathic remedies as well as non-botanical alternative remedies. All rows include a challenged group receiving the studied phytotherapeutic remedy.

The combinations of control groups used in the studies performed under controlled experimental conditions are presented in Figure 4. It was very common to include a challenged and untreated control group (included in 95% of the studies). Placebo was not commonly used, but two studies on cattle and one on poultry included this (27–29).

An unchallenged and untreated control group was included in 79.5% of the studies. Of these, 18 studies (33%) were factorial designs that also integrated a non-challenged group receiving the botanical supplement. The inclusion of a control-group receiving an additional treatment was also relatively common (45%). Often, the choice was the conventional treatment normally used in practice (for example, a coccidiostatic, antimicrobial drug, or vaccination) (36%). However, there were also studies comparing an alternative treatment (15%). These were, for example, supplementing mannanoligosaccharide (*n* = 1), probiotics (*n* = 3), calcium aminosilicate (1), formic acid (1), or a combination of formic acid and copper (1).

### Phytotherapeutic treatments

**Figure 5:**
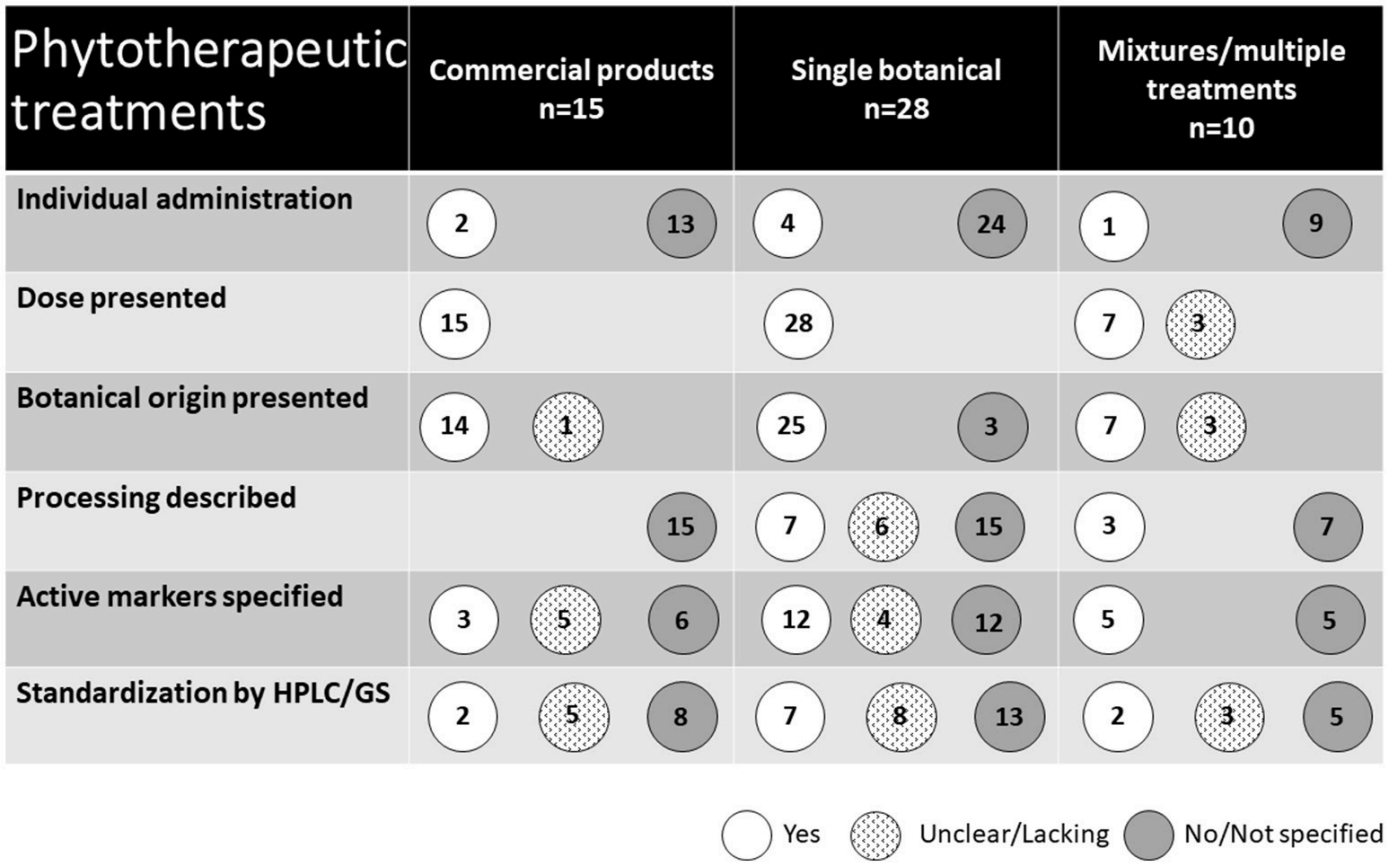
Presentation of the phytotherapeutic remedies used in the 53 studies identified in the review.

The types of phytotherapeutic treatments used are presented in Figure 5. Fifteen studies used commercial treatments available on the market, including products derived from a single botanical as well as botanical mixtures. Of the remaining 28 studies, treatments were derived from a single botanical, although 14 of these included multiple treatment groups receiving different doses. The remaining 10 studies investigated the effect of multiple treatments of single botanicals as well as treatments of mixtures of different botanicals. In total (including commercial products), 12 studies used supplements consisting of several plants. In two of these, the separate effects of the included botanicals were also studied. The majority of the treatments were administered at a group level through feed (*n* = 44) or water (*n* = 2). Five studies used individual oral administration. The remaining two used parenteral and intrauterine administration.

The scientific names of the plants used were given in 34 studies. Sixteen additional studies reported a common name of the plant or a processed plant-derived product. Three studies did not provide any information about the original plant of the naturally derived substance used. Instead, these studies (30–32) reported the use of a purified active substance of natural origin. In total, the use of 12 commercial products was reported in 18 studies. In three of these studies, the commercial product was mixed with an unknown combination of other botanicals.

The type of botanical preparation used varied and in three cases is not stated at all. In eight studies, combinations or several types of preparations were studied, commonly a dried herb extract and an essential oil. Extracts (including dry extracts) were used in 13 studies, essential oils in nine studies, powders and dried ground botanical parts in nine studies. The specification of content or composition of ingredients and dosage of the applied botanicals in the studies reviewed was generally poor. In many cases (37 studies), underlying information about processing, apart from original plant and/or manufacturer, was not given. Twenty studies reported the content of active markers, although a description of the method for establishing this was lacking in all but 11 studies. Full chemical characterization using state of the art analytical method HPLC was carried out in four studies.

### Efficacy of the remedies

Only one study reported no difference between the treated group and the challenged control (category D). However, the results of about half of the studies (*n* = 31) were categorized as uncertain (category C). Seven of these were on cattle, 12 on poultry and 12 on pigs. The reason for this classification varied. In 10 studies, no animals or only a small number of animals showed clinical signs and/or the mortality was very low, leading to questions of whether any of the animals studied were in fact unhealthy. It is possible that they were not affected by the same pathogen/condition, or that the experimental infection was not successful. In four studies, non-specific changes in blood, microflora, or production parameters were discovered, but the importance of these changes on the course of the disease was not clear. Two studies described a reduction of the pathogen in the intestine, but in one of these studies the effect on the clinical disease was not clear. In the other, a large numerical reduction in the number of pathogens was observed. However, no calculations of significance were provided and the sample size was very small. Four studies reported occasional small but significant differences between the treated group and the control group. However, as the significant differences noted were small and inconsistently seen in occasional measurements across a large number of measurements and measuring points, they were classified as uncertain. Three studies observed confounding variables. In two studies, the animals were also vaccinated; leading to difficulties in interpreting to what extent the botanical and the vaccine affected the results.

In 10 studies (19%), the botanical treatment led to a significant decrease in clinical signs or mortality (category A), and in 11 studies (21%) significant positive effects on subclinical measures were noted (category B). In eight of these studies, the effectiveness of the phytotherapeutic was compared to a control group receiving conventional treatment. Of these studies, four were of coccidiosis and the conventional treatment was a coccidiostatic. The botanical treatments were in all cases less effective at reducing lesions than the conventional treatment. Three of the conventional treatments were antibiotics against Mycoplasma gallisepticum (33) and a combination of coccidiosis and necrotic enteritis [two studies from McDougald et al. (20)]. In these studies, the botanical treatment was comparable to the antibiotic treatment.

## Discussion

One approach to resolving contradictory study results is to conduct systematic reviews and metanalyses of rigorous studies (34). However, in the field of alternative medicines, systematic reviews are helpful for sorting through conflicting evidence and exposing the different challenges and limitation that have to be addressed.

From the review, there are some key facts that have been neglected in the study of the efficacy of phytotherapy. The majority of the identified scientific studies showed limitations in the study design of the trials as well as regarding presentation and standardization of the botanical remedies studied. The efficacy of alternative treatments should be scientifically proven using an appropriate study design, particularly Randomized Controlled Trials (RCTs) that are considered the gold standard for estimating the true effect of an intervention. Evidence-based recommendations are primarily related to the clinical design. Undoubtedly, trials should be designed according to the important guidelines/aspects of alternative medicine therapies and frame in it into the definitions of either a veterinary medicinal product or a feed additive, considering the possible use of specific claims (14). But, as in studies of the association of antibiotic exposure and antibiotic resistance, both are subject to common obstacles including control group selection and measures of outcomes (where there is probably a high variability of factors affecting the outcome on the farm). Besides the study design, the correlation structures of the different outcomes and structural characteristics of the farm system in the outcome are challenges for the epidemiological model building process. This heterogeneity of the study design and outcome measures makes them difficult to compare.

Although numerous studies have demonstrated efficacy *in vitro*, respective experimental *in vivo* evidence is still quite limited (35). No general statement about the efficacy of phytotherapeutic products can be validated, as the studies in the review were also lacking reproducibility. A fundamental problem in the clinical research into herbal medicines is whether different products, extracts, or even different lots of the same extract are comparable and equivalent (36). Quality control of botanicals poses significant challenges, as small differences in genetics, soil, temperature, moisture, and time of harvesting can lead to significant differences in the concentration of important constituents (37). Only in a very few cases in the review was the same botanical remedy used to treat the same condition in multiple studies. Mostly, this was seen in publications containing more than one study, but there were also some cases where a similar compound was used in different studies by different authors. For example, Drăgan et al. (29) and Drăgan et al. (38) both present treatment with powder derived from Artemisia annua but, as no description of the process, active markers or manufacturer was given in Drăgan et al. (29), it was not possible to establish the similarity of the botanical product. New analytical methods may possibly be used to establish a “characteristic chemical fingerprint” of chemical ratios that allows a control of consistency between batches (39). These methods may also increase the possibility of identifying contamination, like for example heavy metals, pesticides, wrong plants, and synthetic drugs, which are critical to ensure safe use of botanicals.

Standardization is also difficult for phytotherapeutic products, as they are complex combinations of several phytochemicals. If it is difficult to demonstrate appropriate activity for a single product, it is more complicated with a combination. The specific function of several phytochemicals as well as how they interact with other molecules are still largely unknown, which makes standardization difficult (40–42). In this way, pooling studies that use different herbal products or qualities in a quantitative metanalysis can be misleading (36).

Managing animal health on many farms can be done at different levels of intervention. This approach does not exclude the stringent high-quality research of phytotherapy in research trials, but interventions with phytotherapy must be validated and must be replicable with the overview of efficacy and safety in the farm practice. To overcome obstacles, the need for improved study designs for clinical trials was given priority on a workshop of experts that addressed the current societal challenges of the use of phytotherapy on livestock (43) in order to prove the efficacy of remedies and to implement a monitoring system which enables the assessment of the effectiveness of treatments in farm practice. From the review, no trials have been repeated under the same or comparable conditions (with respect to species, remedy, context/disease, and expertise). Current trials on efficacy were conducted under standardized conditions and do not consider the specific situation which farm animals experience without accounting for the interaction between animals and their living conditions and animals' differing ability to react to certain pathogens.

The knowledge of the essential prerequisites for ensuring a therapeutic effect when treating farm animals under practical conditions does not exist to a sufficient degree in any therapy tool. The efficacy of any action to promote health on the farm, including the use of herbal remedies, cannot be limited to the concentration of pharmaceutical active ingredients alone and cannot be isolated from the context in which it is used, since it is highly farm context-variant. The appropriateness of phytotherapy also depends to a high degree on the appropriateness of initial and boundary conditions on the farm and health management intervention. These conditions are, however, not restricted to phytotherapy, but apply also to conventional medicine and are not commonly met.

**Figure 6:**
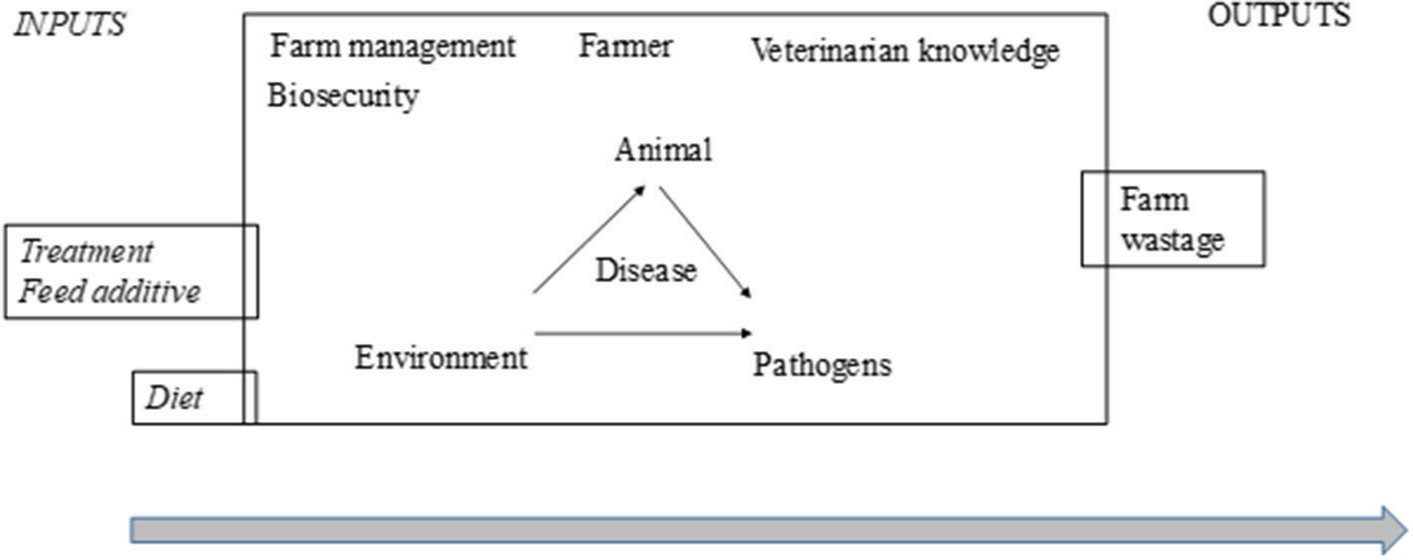
Variability accumulated in the different components or actors when using phytotherapy in farm practice. This overview of variation accumulated from the efficacy and effectiveness of phytotherapy could be converted into the prerequisites that are needed for the manageability of phytotherapy as a therapy tool.

The effectiveness of treatments in farm practice is highly context-dependent and depends on several extrinsic factors (Figure 6). It is not possible to draw firm conclusions until these essential preconditions for a target-oriented and effective use of phytotherapeutic products are established. In the case of phytotherapy, prerequisites include a knowledge of ingredients, efficacy in relation to the dose and mechanisms of action and interactions with other treatments (allopathic or phytotherapeutic). There is also a need for expertise, both to implement the treatment procedures necessary to ensure therapeutic success as well as to provide a deeper understanding of the cost-benefit outcome relationship where positives outweigh negatives while keeping to a minimum negative side effects that might compromise the issues of animal health and welfare, food safety, and environmental impact as common goods. These factors could be included in a critical control points-approach (Control points to minimize the variable effect of phytotherapy), since it fits preventive risk management programs with the ultimate goal of increasing animal product safety (one of the criticism against phytotherapy) and protecting public health. A broader perspective when looking at options for reducing the use of antibiotics in practice might set the design of future trials where study applications of combined interventions (different therapy tools) with a holistic and sustainable approach considering the whole farm system are involved.

## Risk of bias

Publication bias may exist, because studies with no identifiable effects may be difficult to publish, which influences the understanding of the scientific progress in the research area. This is one of the major threats to validity in systematic reviews and meta-analyses (44). The success of a systematic review depends on the availability, accessibility, and quality of literature related to the review question.

The pilot searches revealed a very large number of publications although they were not particularly accurate because they included publications that did not fit the purpose, while still probably missing publications that fitted the inclusion criteria. The review would have benefited from the recent opinion of EFSA and EMA in terms of the standard vocabulary to establish the including criteria (14). Studies were excluded from the review of how phytotherapeutic remedies influence general parameters such as growth, mortality, and other physiological changes or effects on immune system and changes in microbiota not associated with clinical symptoms.

## Conclusion

The systematic review highlighted problems of inadequate study design and lack of reproducibility of studies on phytotherapeutic remedies. Based on the results of the review, it is not currently possible to make definitive conclusions on the effectiveness of phytotherapeutic treatments for diseases in farm practice. This does not mean that there is no potential future place for these treatments, and examples of well-designed RCT studies using of modern tools for standardization of the botanical products with positive effects were identified. To gain further insights into the potential use of phytotherapy and therapy success, these well-designed studies also need to be combined with evaluation of effectiveness under heterogeneous farm conditions. Against the background of large number of side effects associated with conventional antibiotic treatments, future studies should consider that the most sustainable strategy for prevention and therapy may combine viable alternative treatments and changes in farm practice.

## Author contributions

The manuscript is based on a review where L-MT, UE, and IB-P participated. The review was co-lead by L-MT while the manuscript was chaired by IB-P. L-MT and IB-P discussed the content and wrote the manuscript with continuous contributions of UE.

### Conflict of interest statement

The authors declare that the research was conducted in the absence of any commercial or financial relationships that could be construed as a potential conflict of interest.
