# Supplementary material for: Systematic Review of Phytotherapeutic Treatments for Different Farm Animals Under European Conditions
Source: Front Vet Sci. 2018 Jun 22;5:140. doi: 10.3389/fvets.2018.00140 (PMC6024023; doi:10.3389/fvets.2018.00140)
Supplement: Supplementary file 1 [file Table_1.DOCX]

**Studies on Dairy cows/heifers**

| **Author** | **Treatment/**  **Prevention** | **Disorder** | **Source**  **of disease** | **Treated**  **animals** | **Control**** | **Study design** | **Measure of Effect** | **Outcome** | **Effect of**  **remedy** |
| --- | --- | --- | --- | --- | --- | --- | --- | --- | --- |
| Heuwieser et  al. (2000) | T | Endometritis | Naturally  infected | Eucalyptus  52 infected  cows | Lotagen  67 infected  cows  PGF2a  65 infected  cows | RCT  Not blinded | Submission rate  Pregnancy rate  Days to first service  Culling due to  infertility | Alternative treatment  (PGF2a) led to higher  submission rate and  shorter interval to service. | Uncertain |
| Hu et al. (2001) | T | Subclinical mastitis  (caused by S.  Aureus) | Naturally  infected | Panax  Ginseng  3 cows  (6 infected  udder  quarters) | Placebo  3 cows  (3 infected  udder quarters) | RCT  Not blinded | Clinical signs (fever)  Milk:  Somatic cell count  Bacteriological  culture  Daily milk production  Blood: WBC, neutrophil  activity  Lymphocyte activity | Treated animals  showed:  Tendency to decreased  SCC and bacterial presence.  Significant increase in:  number of monocytes  and lymphocytes  lymphocytic activity | Uncertain |
| Balcells et al.  (2012) | T | Ruminal acidosis | nduced | Bioflavex  (Citrus  aurantium  and  paradisi)  8 challenged  individuals | No treatment  8 challenged  individuals  (same as  treatment) | RCT  Cross over  design  Not blinded | Ruminal:  microbial  outflow pH  VFA  Lactate concentration  Lactate producing  and consuming  bacteria | Treatment group: Lower  pH drop, Lower proportion  of acetate and butyrate.  Significantly higher titres  of lactate consuming  bacteria (Megasphera  elsdenii). | Yes |
|  |  | Subclinical ruminal  acidosis | Monitoring  of  disease  incidence in healthy  animals | Bioflavex  (Citrus  aurantium and  paradisi)  24 individuals  fed high  risk diet | No treatment  24 individuals  fed high risk  diet | RCT  Not blinded | All animals:  ADG  Feed consumption  Feed conversion ratio  Canulated (8  cows/group):  Ruminal pH VFA content  Lactate concentration  Lactate producing and consuming  bacteria  Ruminal microbial  outflow | No effect on ADG or  feed consumption.  Cannulated treated | Uncertain |
| Benchaar et  al., (2007) | P | Subclinical ruminal  acidosis | Monitoring  Of disease  incidence | Eugenol  4 low  concentrate  diet 4 high  concentrate  diet | No treatment  4 low concentrate  diet  4 high concentrate  diet | RCT  Not blinded | DMI  Ruminal:  pH  Digestibility  Fermentation  Ammonium nitrogen  balance  Protozoal counts | Treatment had no effect  on any parameters. | Uncertain |
| Bulls |  |  |  |  |  |  |  |  |  |
| Devant et al.  (2007) |  | Subclinical ruminal  acidosis | Monitoring  of  disease  incidence | Biostar  (Cynarin,  Siberian  ginseng,  fenugreek)  30 bulls  fed high  conentrate  diets | No treatment  30 bulls fed  high conentrate  diets  Antibiotic  treatment  (Monesin)  30 bulls fed  high conentrate  diets | RCT  Not blinded | Individual BW  Pen feed consumtion  Ruminal:  pH  VFA content Ammonia/  nitrogen  concentration  Blood:  Cortisol  Insulin  Leptin | BW of animals treated  with antibiotic sign higher  than control group. No  difference between botanical  treatment and  other groups.  Average ruminal pH was  higher in control group  but both treatments  significantly increased  ruminal propionate.  Treatment with plant  extract and antibiotics  gave similar effects on  ruminal and blood parameters  compared to  control. | Uncertain |
| Dairy calves |  |  |  |  |  |  |  |  |  |
| Oliveira et al.,  (2010) | P | Diarrhea (and  other health parameters) | Monitoring  of  disease  incidence | Pomegranate  seed  extract  Low  dose 22  calves  High  dose 22  calves | No treatment  23 calves | RCT  Not blinded | Incidence and  duration of health  disorders (diarrhea,  coghing,  behavioural  changes, increase  in body temperature)  Feed intake  BW  Blood:  Total protein  IgG  Metabolites  Neutrophil phagocytosis  WBC  TNFa  Fecal:  Consistency  Digestibility of  nutrients | Treated calves: reduced  BW gain after day 30 (-  1,8 kg low dose, -4,3 kg  high dose)  No differences in fecal  scores, fever, neutrophil  activity, TNF alpha between  groups.  Treatment cost tended  to increase in treated  group (p<0.10).  Treatment increased the  production of lymphocyte  derived cytokines  from blood mononuclear  cells and IgG increased  faster after vaccination. | Uncertain |
| Bampidis et al.  (2006) | T | Diarrhea | Naturally  infected | Oregano  16 infected  calves | Antibiotic  treatment  (neomycin)  14 infected  calves | RCT  Not blinded | Mortality  Days with atypical  fecal score | No differences between  treatments | Uncertain |
| Bednarek et al.  (2002) | T | Pneumonia | Aseptically  induced | Unicaria  tomentosa  10 challenged  calves | Placebo  10 challenged  calves | RCT  Not blinded | Clinical symptoms  Rectal temperature  Blood:  WBC  Other:  Inflammatory  markers in bronchial  alveolar lavage | Treated group had:  • Significantly lower  body temperature than  control.  • Significantly higher  levels of lymfocytes in  end of study  • Lower levels of neutrophils  initially. | YES |

Annex II: Studies on poultry

| **Author** | **Disorder** | **Source**  **of disease** | **Treated**  **animals** | **Control** | **Study design** | **Measure of Effect** | **Outcome** | **Effect of**  **remedy** |
| --- | --- | --- | --- | --- | --- | --- | --- | --- |
| Arshad et al. (2008) | Infection Escherichia  coli | Experimental  infection at  day 4 | Peganum harmala  15 challenged | No treatment  9 unchallenged  15 challenged | RCT  Not blinded | Clinical signs  Mortality  Necropsy 6 dpi:  Pathological changes  Reisolation of bacteria  Blood 6 days post  infection: GOT,LDH,  ALP, protein, albumin  and globulin | • No statistically significant differences.  • Numerically higher mortality and reisolation frequency in treated birds compared to positive control. | Uncertain |
|  | Infection Escherichia  coli | Experimental  infection at  day 15 | Peganum harmala  6 challenged | No treatment  9 unchallenged  7 challenged | RCT  Not blinded | Clinical signs  Mortality  BW  Necropsy 6 dpi:  Pathological changes  Reisolation of bacteria  Blood 6 days post  infection: GOT,LDH,  ALP, protein, albumin  and globulin, erythrocyte and granulocyte count | Treated birds compared to challenged control:  • Similar mortality.  • Surviving chickens recovered significantly faster.  • Significantly lower bacterial recovery/g tissue.  Positive control:  • Total granulocyte count  significantly higher than nonchallenged  control and treatment.  BW of treated birds higher than positive control, lower than negative control. | Yes |
| (Baurhoo  et al. (2007) | Infection Escherichia  coli | Experimental  infection at day 29 | Lignin  Low dose  12 challenged  12 unchallenged  High dose  12 challenged  12 unchallenged | No treatment  12 challenged  12 unchallenged  Antibiotic (virginiamycin)  12 challenged  12 unchallenged  Other treatment  (mannanoligo saccharide)  12 challenged  12 unchallenged - | RCT  Not blinded | BW  Feed intake  Feed conversion  Necropsy d 3, 6, 9 (2  birds/group):  Cecal content:  Microbial count &  serotyping of E. coli | • No significant differences in production parameters between groups.  The lignin treatment:  • Reduced the population of E. coli, lactobacilli and  bifidobacteria | Uncertain |
| Stipkovits  et al. (2004) | Mycoplasma  gallisepticum | Experimental  infection on  day 21 | Fermented  wheat germ  extract  30 challenged | No treatment  30 challenged  30 unchallenged  Antibiotic  (tiamulin)  30 challenged | RCT  Not blinded | Clinical signs  Necropsy d 9 pi:  Gross lesions  Histological lesions  Presence of challenge strain in organs  Blood:  Specific antibodies | Treatment group and group treated with antibiotics remained clinically healthy when  challenged control developed clinical symptoms. They also had significantly lower lesion scores and increased BW compared to challenged untreated  individuals | YES  (Comparable  to antibiotic) |
| Christaki  et al. (2004) | Coccidiosis  (E. tenella) | Experimental  infection on  day 14 | Apacox***  Low dose  30 challenged  High dose  30 challenged | No treatment:  30 unchallenged  30 challenged  Coccidiostatic:  (lasalocid)  30 challenged | RCT  Not blinded | Clinical signs (bloody  diarrhea)  Mortality  BW  Feed conversion  Oocyst count  (Day 7,14,20-26)  Cecal lesion (9/group on 7 dpi) | Treated birds had:  • Lower intensity of diarrhea and lower oocyst shedding comp to challenged control.  • High dose led to lower oocyst shedding than low dose.  • More symptoms and lesions compared to group treated with anticoccidia  • Improved production parameters compared to challenged control but lower than non-challenged control and  coccidostatic group. | YES (but not  as efficient as  coccidiostatic) |
| Dragan et  al. (2010) | Coccidiosis  (E. tenella) | Experimental  infection on  day 10 | Artemisia annua  Oil  20 challenged  Powder  20 challenged  Pimpinella  anisum  oil  20 challenged  Combination  20 Challenged | No treatment:  20 unchallenged  20 challenged  Placebo:  20 challenged  Coccidiostatic  (Lasalocid)  20 challenged | RCT  Not blinded | BW  Feed conversion  Mortality  Clinical signs  Fecal  oocyst shedding/g  Necropsy 7 dpi  (10 birds/group)  Lesion score | Supplementing with Artemisia annua:  • Led to numerically lower mortality and increased weight gain compared to  challenged untreated control and placebo (although not as efficient as coccidiostatic and unchallenged group).  • Significantly reduced oocyst shedding compared to positive  control and placebo group. Powder was most effective.  Lesion score:  • Was low in all groups but information about number of  individuals is lacking  Mortality was low in all groups  but occurred after d17 in positive  control (2/10), placebo  (1/10) and P. anisum (1/10). | Yes (but not as efficient as  coccidiostatic) |
| Giannenas  et al.  (2003) | Coccidiosis  (E. tenella) | Experimental  infection on  day 14 | Origanum vulgare  subsp.  hirtum  30 challenged | No treatment  30 unchallenged  30 challenged  Coccidiostatic  (Lasalocid)  30 challenged | RCT  Not blinded | BW  Feed consumption  Mortality  Clinical signs (diarrhea)  Feces:  Oocyst shedding  Lesion score 7dpi | Treated birds showed:  • Performance parameters  comparable to non- challenged  control.  • Less severe symptoms of  diarrhea, less severe lesion  scores, lower mortality and  lower levels of oocyst shedding  than challenged control.  Birds fed coccidiostatic:  • Exceeded treatment group in  all measures | YES  (not as good as coccidiostatic  but comparable  to no infection) |
| Matthews & Southern,  (2000) | Coccidiosis  E. acervulina | Experimental  infection  1. Low  (chronic)  dose on  day: 1,4,7  & 10  2. High  (acute)  dose day 1 | Betaine  1. 50 challenged  2. 50 challenged  50 unchallenged | No treatment  50 challenged  (1)  50 challenged  (2)  50 unchallenged | RCT  Not blinded | DWG  Feed intake  Lesion score  Blood d 7 & 14 dpi:  Total carotenoids  Total protein | Treatment with betaine:  • Increased overall ADG in chronically infected birds but tended to decrease it in uninfected birds (p<0,09).  • Plasma total protein on day tended to increase in low dose infected birds (p<0,06) and decrease in uninfected birds (p<0,01). | Uncertain |
|  | Coccidiosis  E. acervulina | Experimental  infection  1. Low dose  on day:  1,4,7 & 10  2. High dose  day 1 | Betaine  1. 100 challenged  2. 100 challenged  100 unchallenged | No treatment  100 challenged  (1)  100 challenged  (2)  100 unchallenged | RCT  Not blinded | DWG  Feed intake  Lesion score 14 dpi  Blood:  Total carotenoids  Total protein | Treatment with betaine:  Significantly increased lesion score (p<0,03) in infected birds.  Plasma total protein and carotenoids  increased on d 14 in low dose infected birds  (p<0,04) and carotenoids  increased in high dose infected birds (p<0,05). | Uncertain |
| Oviedo-  Rondon et  al. (2006) | Coccidiosis  (E. tenella) | Experimental  infection  Day 19 | Crina Alternate  (CA)  36 challenged  CA+ vaccination  36 challenged  Crina Poultry  (CP)  36 challenged  CP+vaccinatio  n  36 challenged | No treatment  36 unchallenged  36 challenged  Vaccination  36 challenged  Antibiotic  (Bacitracin +  Monesin)  36 challenged | RCT  Not blinded | BW  FI  FCR  Mortality  Lesion score  7 dpi (12 birds/group)  Oocyst count 7 dpi (2  pooled samples/6  birds = 12 samples/ group)  Calculated anticoccidiostatic index | Non vaccinated birds fed CA, vaccinated control and vaccinate birds fed CP had significantly better BWG during study period compared to infected control. There were no significant differences between  vaccinated treatment groups and uninfected control. No treatment affected mortality. CA treatment maintained BWG and FCR similar to uninfected control. No differences in overall lesion score between treatments and botanical supplementation did not significantly reduce oocyst shedding compared to negative  control | Uncertain |
| Dragan et al., (2014) | Coccidiosis  E. tenella | Experimental  infection  Day 10 | Artemisia annua leaf powder  (AAp):  20 challenged  Artemisia annua & Foeniculum  vulgare  essential oils  (EO):  20 challenged | Not treatment:  20 challenged  20 non challenged  Coccidiostatic  (Lasalocid)  20 challenged | RCT  Not blinded | Clinical signs  Mortality  Weight gain  Feed conversion  Fecal:  Score  Oocyst output to 35  dpi.  Lesion score 7 dpi  (10 birds/group?) | Mortality only occurred in positive  control group (7/20).  AAp decreased severity and frequency of clinical symptoms and significantly reduced fecal shedding compared to challenged control.  EO also reduced clinical signs and oocyst shedding although not as much. Lasalocid treated chickens performed best in all measured categories. | YES  (But coccidistatic  More effective) |
| Hassan et  al. (2008) | Coccidiosis  E. tenella | Experimental  infection d  10 | Gyamopsis  tetragonoloba  (guar meal)  30 challenged  30 unchallenged | No treatment  30 challenged  30 non challenged | RCT  Not blinded | BW  Feed conversion  Mortality  Fecal:  Oocyst output  Bloody diarrhea | Treated group had:  No cases with bloody diarrhea compared to 5 cases in the non-treated challenged group. A significantly lower oocyst output on day 7 pi and d 10 pi compared to non-treated challenged group. No effect on BW and feed conversion ratio 11 dpi. Low mortality in all groups indicated mild infection. | Uncertain |
| Lee et al.  (2008) | Coccidiosis  E. Acervulina | Experimental  infection day  12 | Prunus salicina  0,5% of diet  10 challenged  1% of diet  10 challenged | No treatment  10 challenged  10 unchallenged | RCT  Not blinded | BW  Oocyst shedding  Spleen lymphocyte  proliferation 10 dpi  Intestinal expression  of INFg and IL15 10  dpi | 1% diet and not challenged group had significantly increased body weight compared to 0,5 % group and challenged control. Oocyst shedding was significantly reduced in 1% group. 0,5 % group had significantly increased lymphocyte proliferation and the 1 % group had significantly increased expression of IFN and IL10. | Yes |
| Allen  (2003) | Coccidiosis  Mix  (acervulina,  tenella,  maxima,  necatrix) | Experimental  infection at 4  weeks | Echinacea  purpurea d1 to  2 weeks:  0,1% of diet  10 challenged  10 unchallenged  0,1% + vaccination  10 challenged  10 unchallenged  0,5%  10 challenged  10 unchallenged  0,5% + vaccination  10 challenged  10 unchallenged | No treatment  10 challenged  10 unchallenged  Vaccination  10 challenged  10 unchallenged | RCT  Not blinded | BW  Necropsy 10 dpi:  Lesion score  Plasma:  Carotenoids  Nitrite/nitrate | Echinacea in combination with  vaccination protected against  weight gain suppression and  development of lesions. Supplement  in itself did not give  significant effect against challenge. | Yes |
| Mcdougald  et al.  (2014) | Coccidiosis  E. acervulina  and E. maxima | Experimental  infection at  14 days | Muscadine  pomace  2% of diet  24 challenged  5% of diet  24 challenged | No treatment  24 challenged | RCT  Person  scoring  lesions  blinded to  treatment | BW  Feed conversion  Mortality  Necropsy 7 dpi  Lesion scoring | • Lesion scores of E. maxima and E. acervulina were significantly  reduced by treatment  compared to infected control.  No difference in weight gain.  • No mortality in study population. | Yes |
|  | Coccidiosis  E. acervulina and E. maxima | Experimental  infection at 21 days | Muscadine  pomace +Vaccination:  0,5% of diet  8 unchallenged  36 challenged  2% of diet  8 unchallenged  36 challenged | No treatment  ? unchallenged ? challenged  Vaccination  ? challenged  ? unchallenged | RCT  Person scoring  lesions  blinded to  treatment | BW  Feed conversion Mortality  Necropsy 6 dpi  Lesion scoring | Combination of vaccine and supplement significantly decreased lesion scores compared to infected vaccinated control.  • Weight gain post challenge was numerically improved in  birds receiving supplement compared to vaccinated control. | Uncertain |
|  | Coccidiosis  and necrotic  enteritis  E. acervulina,  E. maxima and  Cl. perfringens | Experimental  infection at  day 14 | Muscadine  pomace  0,5% of diet  90 challenged  2% of diet  90 challenged | No treatment  90 challenged  90 unchallenged  Antibiotic  (bacitracinmethylene  disalycilate)  90 challenged | RCT  Person  scoring  lesions  blinded to  treatment | BW  Mortality  Feed conversion  Necropsy 28 dpi  Lesion scoring | • Mortality was numerically lowered compared to nontreated  control in all treatment  groups.  In 2 % and antibiotic groups:  • Lesion score in surviving birds was significantly lower compared to challenged control.  • Weight gain in surviving birds significantly increased. | Yes |
|  | Coccidiosis  and necrotic  enteritis  E. acervulina,  E. maxima and Cl. perfringens | Experimental  infection at  day 14 | Muscadine  pomace  0,5% of diet  80 challenged  2% of diet  80 challenged | No treatment  80 challenged  80 unchallenged  Antibiotic  (virginiamycin)  80 challenged | RCT  Person  scoring  lesions  blinded to  treatment | BW  Mortality  Feed conversion  Necropsy 22 dpi  Lesion scoring | Mortality in all treatment  groups was significantly lowered compared to non-treated control.  Supplement and antibiotic groups had:  • Lower lesion scores comparable  to non-challenged control  and significantly lower than challenged control.  • Numerically improved weight gain and significantly improved feed conversion. | Yes |
| McReynol  ds et al.  (2009) | Necrotic enteritis  Cl. perfringens | Experimental  infection  (and immunocompromise)  at d 14 | Herbal blend  A  50 challenged  B  50 challenged  C  50 challenged | No treatment  50 challenged  50 unchallenged  Probiotic  treatment  D  50 challenged  E  50 challenged | RCT  Not blinded | Mortality (all animals)  Lesion score  (24 /treatment group)  Intestinal content Cl.  Perfringens  (10 animals/ treatment  group) | Compared to positive control:  • A, C, D, E significantly decreased lesion scores  • A and D significantly reduced mortality  • D had reduced intestinal content of Cl. perfringens. Probiotic treatment D was comparable to non-challenged control and birds in this group performed significantly better than herbal blends. | YES  (But less effective  than probiotic  blend D) |
|  | Necrotic enteritis  Cl. perfringens | Experimental  infection  (and immunocompromise)  at d 14 | Herbal blend  A (as above)  74 challenged  Herbal & probiotic  AD  74 challenged | No treatment  37 challenged  37 unchallenged  Probiotic  treatment  D (as above)  74 challenged | RCT  Not blinded | Mortality (all animals)  Lesion score  (24 /treatment group)  Intestinal content Cl.  Perfringens  (10 animals/ treatment  group) | Compared to positive control:  • Numerical reduction of lesions  in A and D and a significant  reduction in AD.  • Mortality in A, D and AD  were numerically reduced.  • Significant reduction in intestinal  content of Cl. Perfringens  in A, D and AD. | Uncertain |
|  | Necrotic enteritis  Cl. perfringens | Experimental  infection  (and immunocompromise)  at d 14 | Herbal blend  A (as above)  100 challenged  Herbal & probiotic  AD  100 challenged | No treatment  50 challenged  50 unchallenged  Probiotic  treatment  D (as above)  100 challenged | RCT  Not blinded | Mortality (all animals)  Lesion score  (40 /treatment)  Intestinal content Cl.  Perfringens  (10 animals/ treatment group) | Compared to positive control:  • Lesion score for all groups  were significantly lower.  • Mortality in A, D and AD  were numerically reduced.  • Significant reduction in intestinal  content of Cl. Perfringens  in A, D and AD. | Yes |
| Engberg et  al. (2012) | Necrotic enteritis  Cl. perfringens | Experimental  infection day  17-20 | Artemisia Annua  Dried leaves  80 challenged  Extract  80 challenged | No treatment  80 challenged  80 unchallenged | RCT  Not blinded | BW  (40 /treatment)  Lesion score  (15 /treatment)  Intestinal content:  (15 /treatment)  Cl. Perfringens  pH Ileal and cecal microflora | Group treated with extract had:  • Significantly lower lesion scores compared to other challenged groups.  • Higher weight gain than other challenged groups.  • Extract lowered the cecal number of Cl. Perfringens compared to other challenge groups. No differences in ileal content between groups. | Yes |
| Mitsch et  al. (2004) | Necrotic enteritis  Cl. Perfringens | Natural infection  Incidence  from d 1-  slaughter | Herbal blend  A  92 000 birds  6 flocks  B  113 800 birds  6 flocks | No treatment  208 700 birds  12 flocks | RCT  Not blinded | Fecal content of Cl.  perfringens:  5-10 samples/flock  Intestinal content of  Cl. perfringens  9 birds/flock  Necropsy  (Intestinal lesions) (only of birds from  flocks with increased  mortality) | • Control group consistently showed highest concentration of Cl perfringens in feces and intestine as well as a greater percentage of samples positive for Cl perfringens. No difference between A and B.  • No clinical necrotic enteritis in treated groups while doubled mortality rate on day 14-16 with lesions associated with  necrotic enteritis occurred in 2  control flocks. | Uncertain |
| Gowda,et  al. (2008) | Mycotoxicosis  Aflatoxin | Experimental  challenge  from day 1. | Curucuma  longa (CL)  20 challenged  20 unchallenged  Curucuma  longa (CL) +  calcium aminosilicate  (CA)  20 challenged | Calcium aminosilicate  20 unchallenged  20 challenged  No treatment  20 unchallenged  20 challenged | RCT  Not blinded | BW d 21  Feed intake  ADG  Mortality  Liver (8 birds/group):  Weight  Antoxidant status  Histopathology  Blood (8 birds/group):  Total protein  Albumin  Cholesterol  Uric acid  Gamma glutamyl  transferase | In challenged birds CL treatment led to  • significantly (p<0,05) improved weight gain compared to challenged control.  All treatments decreased:  • Changes in blood serum and the severity of hepatic lesions in liver compared to challenge  control.  Birds given CA:  • Performed comparable to unchallenged control and  better than birds supplemented CL.  Very low mortality in the study. | Yes  (but not as  effective as  CA) |
| Gowda et  al, (2009) | Mycotoxicosis  Aflatoxin | Experimental  challenge  day 1 | Curucuma  longa (CL)  High dose  30 challenged  30 unchallenged  Medium dose  30 challenged  Low dose  30 challenged | No treatment  30 challenged  30 unchallenged | RCT  Not blinded | BW d 21  Feed intake  ADG  Mortality  Liver (12  birds/group):  Weight  Antioxidant status  Blood (12  birds/group): glucose,  total protein, albumin,  globulin, g-glutamyl  transferase, aspartate  amino- transferase,  uric acid and Ca | Low dose and medium dose significantly improved weight gain compared to challenged  control.  All levels of supplement significantly lowered the change in  relative liver weight seen in  challenged control as well as  the decrease in antioxidant functions due to AFB1.  Medium dose ameliorated the  adverse effects of AFB1 on  serum chemistry (total protein,  albumin and g-glutamyl transferase  activity) compared to  other challenged groups.  Low mortality during study  period (2 from challenged  control and 1 from group fed high dose of treatment) |  |
| Revajova  et al.  (2013) | Mycotoxicosis  Fusarium toxins  (deoxynivalenol  & zearalenone) | Contaminated  feed from  week 2Contaminated  feed from  week 2 | Lignin  20 challenged  20 unchallenged | No treatment  20 challenged  20 unchallenged | RCT  Counting  of duodenal  lymphocytes  was blinded  to treatment  identity. | Clinical signs  Mortality  At 4 weeks of age:  (6 birds/group)  Blood:  WBC  Phagocytic activity  IgM bearing cells  Duodenal sample:  CD4+ cells | No clinical signs or mortality during the study period. Treatment did not affect WBC, phagocytic activity or IgM bearing cells.  A tendency that lignin prevented  a reduction in duodenal  CD4+ cells but the effect was not significant when compared to both challenged and unchallenged control. | Uncertain |
| Laying hen | | | | | | | | |
| Gresakova  et al.  (2012) | Mycotoxicosis  (Zearalenone) | Experimental  challenge on  day 14 | Lignin  20 challenged  20 unchallenged | No treatment  20 challenged  20 unchallenged | RCT  Not blinded | Clinical signs  At 6 weeks of age  (8 chickens/group)  Blood glutathionperoxidase,  Hb,  superoxiddismutase  activity, activity of  thioredoxin reductase  GGT, alphatocopherol,  retinol conc.  Gluthatione peroxidase  (GPx) activity  in:  Liver  Kidney  Duodenal mucosa | • No clinical signs of mycotoxicosis  throughout study period.  • Only effect of lignin was a  prevention of increase GPx  activity in the duodenal mucosa  associated with challenge  of toxins. | Uncertain |
| Ordonez  et al.  (2008) | Salmonellosis  Salmonella  enterica | Experimental  infection at  19 weeks of  age | Eugenia caryophyllata  (Syzygium  aromaticum)  25 challenged  25 unchallenged | No treatment  25 challenged  25 unchallenged | RCT  Not blinded | Bacteriological culture  weekly:  Feces  Eggs  Necropsy  15 dpi  5 hens/group  Signs of systemic  infection  Culture of samples  from liver, spleen,  ovary, ceca  29 dpi  5 hens/group  Signs of systemic  infection  Culture of samples  from liver, spleen,  ovary, ceca  Blood  15 dpi &  29 dpi  (5 birds/group)  Antibodies | Treated and not treated challenged group shed bacteria in  feces in a similar manner.  The differences between challenged  treatment and control  were: 29 days post infection challenged  treatment group was  negative on bacterial culture from liver, spleen and ovary whereas challenged control  was positive.  On day 30 eggs from treated group tested negative and the control group was positive. | Uncertain |
| Cacho et  al. (2010) | Coccidiosis  Eimeria tenella | Experimental  challenge at  4 weeks of  age | Artemisia Annua  High dose  75 challenged  Low dose  75 challenged | No treatment  75 challenged  75 unchallenged | RCT  Not blinded | Mortality  7 days pi:  5 birds/group:  Lesion score  Oocyst count  Oocyst viability:  (By reinoculation in  healthy birds, sporulation  ate, permeability) | Compared to challenged control treated groups had:  • Significantly reduced oocyst output and lesion scores.  • Significantly lower mortality.  Treatment significantly reduced sporulation rate of oocysts from medicated birds. | Yes |

Annex III: Studies on pigs

| **Author** | **Treatment/**  **Prevention** | **Disorder** | **Source**  **of disease** | **Treated**  **animals** | **Control**** | **Study design** | **Measure of Effect** | **Outcome** | **Effect of**  **remedy** |
| --- | --- | --- | --- | --- | --- | --- | --- | --- | --- |
| Jugl-  Chizzola et  al. (2005) | **P** | Escherichia  coli (E. coli)  infection | Natural  infection  around  weaning | Thymus  vulgaris from 3  days before  weaning  22 pigs | No treatment  22 pigs | RCT  Randomisation  based  on sex and  live weight  Not blinded | BW  Feed intake  Clinical signs  (Behaviour, discharge,  respiration,  coughing, diarrhea)  Rectal swabs from  pigs with diarrhea  (n=1):  Bacteriological culture  of haemolytic E.  coli. | No differences in weight  gain or feed efficiency between  groups.  One pig from control group  suffered from diarrhea. No  other animals showed  symptoms of disease. | Uncertain |
|  | **P** | Escherichia  coli (E. coli)  infection | Natural  infection  around  weaning | Thymus  vulgaris  from 3 days  before weaning  17 pigs | No treatment  18 pigs  Antimicrobial  (Flavophospholipol)  18 pigs | RCT  Randomisation  based  on sex and  live weight  Not blinded | BW  Feed intake  Clinical signs  (Behaviour, discharge,  respiration,  coughing, diarrhea)  Rectal swabs  (7 samples from 12  pigs/group + pigs  with diarrhea):  Bacteriological culture  of haemolytic E.  coli. | No differences in weight  gain or feed efficiency between  groups.  One pig from thymus vulgaris  group suffered from diarrhea.  No other animals  showed symptoms of disease.  Hemolytic E. coli was recovered  from:  10 samples in thymus vulgaris  group. 16 samples  from control and 13 samples  from group treated with  antimicrobial. | Uncertain |
| Liu et al.(2013a) | **P** | Escherichia  coli (E. coli)  infection | Experimental  challenge  at 25 days  of age | Capsicum  oleo- resin  (CO)  8 challenged  8 nonchallenged  Garlic botanical  (GB)  8 challenged  8 nonchallenged  Turmeric oleoresin  (TO)  8 challenged  8 nonchallenged | No treatment  (NT)  8 challenged  8 nonchallenged | RCT  (block design  with  weight within  sex as  block)  Not blinded (uses “sham  group” but  does not  specify  blinding  procedure) | BW  Clinical signs  Fecal score  Fecal sample:  day 0,3,5 (all pigs),  day 8,11 (4 pigs/group)  Bacteriological culture  Histopathology:  (day 5: 4 pigs/group,  day 11: 4  pigs/group)  Blood  (day 0,5,11)  WBC, TNFa, Creactive  protein,  haptoglobin. | All treatments significantly  reduced frequency of diarrhea  and the diarrheal  scores in challenged and  non- challenged groups.  Treatment significantly lowered the increase in neutrophils,  TNFa, c-ractive protein  and haptoglobin as well  as histopathological changes  and increased recruitment  of machropages in  ileal villi seen in challenged  control.  No effect of treatment on  the fecal presence of coliforms was reported | **Yes** |
| Manzanilla  et al. (2004) | **P** | Escherichia  coli (E. coli)  infection | Natural  infection  (stress  induced) | Oregano, Cinnamon  and  Mexican pepper  (OCM)  Low dose  36 pigs  High dose  36 pigs  Lowdose +  formic acid  36 pigs  High dose+  formic acid  36 pigs | No treatment  36 pigs  Formic acid  36 pigs | RCT  Not blinded | FI  Incidence of diarrhea  Mortality  Necropsy d 24 & 25  (  1 pig/group)  pH, morphology,  microbial proliferation,  hindgut fermentation. | N.B! All pigs were treated  with antibiotic for diarrhea.  OCM significantly increased  intestinal number of lactobacilli  and decreased total  microbial mass.  Mortality was low but 4 out  of 5 pigs with diarrhea were  from the groups not supplied  with OCM. | Uncertain |
| Sads &  Bilkei,  (2003) |  | Escherichia  coli (E. coli)  and Haemophilus  parasuis  infection | Naturally  infected | Origanum vulgare  (Oregpig)  312 pigs vaccinated  against  E. coli and H.  parasuis  309 pigs vaccinated  against  H. parasuis | No treatment  308 pigs vaccinated  against  E. coli and H.  parasuis  301 non vaccinated  pigs | RCT  Not blinded | ADG  FI  Mortality due to E.  coli or H. parasuis  (diagnosed by  pathological changes  and  reisolation of pathogen  at necropsy) | Mortality in non-treated,  non-vaccinated group was  significantly higher than  other groups.  Groups given Oregpig  showed significantly increased  BW on d 35 and  improved ADG compared to  other groups. | Uncertain |
| Turner et al.  (2002b) |  | Salmonellosis  (Salmonella  enterica  serovar  typhimurium) | Experimental  challenge  on day 14 | Quillaja Saponaria  Low dose  12 challenged  pigs  12 unchallenged  pigs  Medium dose  12 challenged  pigs  12 unchallenged  pigs  High dose  12 challenged  pigs  12 unchallenged  pigs | No treatment  12 challenged  pigs  12 unchallenged  pigs | RCT  Random allotment  blocked by  weight and  equalized for  sex  Not blinded | ADG  FI  Rectal temperature  Fecal shedding  d 7 and 14 pi.  Blood d 7 pi:  (6 pigs/group)  haptoglobin, O1-  acid glycoprotein  (AGP), immunoglobulin  M (IgM),  and immunoglobulin  G (IgG)  IGF-I  Phagocytic assay | No differences in measured  parameters were reported. | **No** |
| Turner et al.  (2002a) |  | Salmonellosis  (Salmonella  enterica  serovar  typhimurium) | Experimental  challenge  on day 14 | Ascophylum  nodosus  Low dose  12 challenged  pigs  12 unchallenged  pigs  Medium dose  12 challenged  pigs  12 unchallenged  pigs  High dose  12 challenged  pigs  12 unchallenged  pigs | No treatment  11 challenged  pigs  11 unchallenged  pigs | RCT  Random allotment  blocked by  weight and  equalized for  sex  Not blinded | Rectal temperature  ADG  FI  Fecal shedding  day 7 and 14 pi.  Blood d 7pi:  (6 pigs/group)  haptoglobin, O1-  acid glycoprotein  (AGP), immunoglobulin  G (IgG),  and immunoglobulin  M (IgM) | No differences in measured  parameters were reported  except for a small tendency  of treatment increasing  growth. | Uncertain |
| Janczyk et  al. (2008) | **P** | Salmonellosis  (Salmonella  enterica  serovar  typhimurium) | Experimental  challenge  at d 29 | Thymol  8 challenged  pigs  8 non challenged | No treatment  8 challenged  8 non challenged | RCT  Not blinded | Jejunal content | Differences in the microflora  of treated and non-treated  pigs were seen. Challenge  with S. typhimurium had  little effect on microbial  community. | Uncertain |
| Van Parys  et al. (2010) | **P** | Salmonellosis  (Salmonella  enterica  serovar  typhimurium) | Experimental  challenge  at  42 days  of age | Castanea sativa  6 challenged pigs | No treatment  6 challenged  pigs  3 non challenged  pigs | RCT  Not blinded | Mortality  Fecal samples:  Bacteriological reisolation  Necropsy 4 dpi:  Bacteriological reisolation | Treatment had no effect  on excretion of pathogen  and a numerical decrease  in colonization.  No mortality except one  pig from treated and  challenged group. | Uncertain |
| Liu et al. (  2013) |  | Porcine  reproductive  and respiratory  syndrome  (PRRS) | Experimental  infection  at 35  days of  age | Capsicum oleoresin  (CO)  8 challenged  8 nonchallenged  Garlic botanical  (GB)  8 challenged  8 nonchallenged  Turmeric oleoresin  (TO)  8 challenged  8 nonchallenged | No treatment  (NT)  8 challenged  8 nonchallenged | RCT  Blocked by  initial BW,  equalized for  sex and  ancestry  Not blinded | Clinical signs (respiratory,  general)  BW d 0, 7 and 14 pi.  Rectal temperature d 4,  7, 9, 11 and 14 pi.  Blood d 0, 7 and 14 pi:  Viral load  Antibody titers  Inflammatory markers | All treatments led to  significantly lower rectal  temperatures on day 4 pi,  decreased viral load,  increased blood lymphocyte  production on d 7,  lowered CRp and proinflammatory  cytokines in  infected pigs.  Mortality was low (3/64)  pigs.  TO improved feed efficiency. | Yes |
| Greiner et  al. (2001) | **P** | PRRS | Experimental  challenge  at  d 29 | Soy bean  Low dose  12 challenged  pigs  Medium dose  12 challenged  pigs  High dose  12 challenged  pigs | No treatment  12 challenged  pigs | RCT  Not blinded | Clinical signs  Body temperature  FI  BW  Blood:  Concentration of virus  Interferon (IFN)  O1-acylglycoprotein  (AGP)  Antibodies:  (Actinobacillus pleuropneumoniae,  Mycoplasma  hyopneumoniae, PRRS  virus, swine influenza,  transmissible gastroenteritis  virus)  Spleen and thymus  weight | Spleen significantly heavier  in supplemented pigs  indicating activation of  immune response.  IFN concentrations lower  and greater AGP response  during high viremia  periods.  Tendency (p<0,07) to  reduced serum concentration  of virus.  No differences in clinical  signs reported. | Uncertain |
| Hermann et  al. (2003) | **P** | PRRS | Experimental  challenge  at  d 29 | Echinacea purpurea  2%  15 challenged  15 unchallenged  4%  15 challenged  15 unchallenged | No treatment  15 challenged  15 unchallenged  Antimicrobial  (carbadox)  15 challenged  15 unchallenged | RCT  Not blinded | BW  ADG  FI  Blood:  Concentration of virus  Development of anitbodies | No significant effect of  Echinacea purpurea  on pig performance,  viremia or development  of antibodies were reported. | No significant effect of  Echinacea purpurea  on pig performance,  viremia or development  of antibodies were reported. |
| Schoene et  al. (2006 | **P** | Post weaning  diarrhea | Natural  infection | Foeniculi aetheroleum  16 pigs  Carvi aetheroleum  16 pigs | No treatment  16 pigs  Formic acid +  copper  16 pigs | RCT  Not blinded | Incidence of diarrhea  FI  BW  Feed efficiency | Diarrhea only occurred in  the group treated with  Foeniculi aetheroleum.  No significant differences  in production parameters | Uncertain |
| Kis & Bilkei,  (2003) | **P** | Endometritis | Natural  incidence | Origanum vulgare  subsp.  Hirtum (Oregpig)  44 primiparous  sows  46 multiparous  sows | No treatment  44 primiparous  sows  46 multiparous  sows  Antimicrobial  (chlortetracycline)  44 primiparous  sows  46 multiparous  sows | RCT  Not blinded | Body condition  FI  Time to estrus  Weaning to estrus  interval | Very few (2,8 %-6,5 %) of  sows failed to show estrus  by 18 days after  weaning.  The weaning to estrus  interval was significantly  shorter in sows fed oregpig.  Farrowing rate for primiparous  sows was significantly  higher for sows  fed oregano. | Uncertain |
| Magi et al.  (2005) | **T** | Pig nodular  worm  (Oesphagost  omum spp.) | Experimental  challenge  at  2 months  of age | 1. Curbita pepo  (pumpkin)  4 challenged pigs  2. Tancaetum  vulgare (tansy)  4 challenged pigs  3. Acorus calamus  (sweet flag)  4 challenged pigs  4. Allium sativum  (garlic)  4 challenged pigs | No treatment  4 challenged  pigs  Antiparasitic  agent  (Ivermectin)  4 challenged  pigs | RCT  Not blinded | Fecal sampling:  Excreted eggs  Necropsy 4 months pi:  Recovery of parasite | All treatments led to a  marked reduction in  shedding of eggs as well  as intestinal worm burden  (78-98 %)  Sweet flag showed highest  effect (98 % reduction  compared to control) and  garlic was least effective. | Uncertain  (Sweet  flag more  effective  than  ivermectin) |
