# Supplementary material for: Systematic Review of Phytotherapeutic Treatments for Different Farm Animals Under European Conditions
Source: Front Vet Sci. 2018 Jun 22;5:140. doi: 10.3389/fvets.2018.00140 (PMC6024023; doi:10.3389/fvets.2018.00140)
Supplement: Supplementary file 2 [file Table_2.DOCX]

Annex IV: Origin and standardization of botanicals

| Author | Manufacturer | Original plant | Identification  of original  plant described | Harvest  conditions | Processing  procedure  described | Active markers/  marker: | Analytical methods**  used for  chemical characterization | Administration |
| --- | --- | --- | --- | --- | --- | --- | --- | --- |
| Poultry | | | | | | | | |
| Mycotoxicosis | | | | | | | | |
| (N. K. S.  Gowda et  al., 2008) | No | Curucuma longa | No | No | Yes | Total curucuminoid  content (curcumin,  bisdemethoxycurcumin,  demethoxycurcumin) | HPLC | Part of feed |
| (N K S  Gowda et  al., 2009) | No | Curucuma longa | No | No | Yes | Total curucuminoid  content (curcumin,  bisdemethoxycurcumin,  demethoxycurcumin) | HPLC | Part of feed |
| (Revajova et  al., 2013) | Institute of  Chemistry, Bratislava,  Slovakia | Waste wood | No | No | No | 15,2 % Methoxyl  groups (of prepared  lignin) | Not specified | Part of feed |
| Gresakova  et al., 2012 | Chemical Institute  of the Slovak  Academy of  Sciences in  Bratislava, Slovakia | Wood | No | No | No | 15,2 % methoxyl  groups  6,5 % phenolic OH | Not specified | Part of feed |
| E. Coli | | | | | | | | |
| Baurhoo et  al (2007) | Alcell Technologies  Inc., Montreal,  Quebec, Canada | Alcell lignin | No | No | No | No |  | Part of feed |
| (Arshad et  al., 2008) | No | Peganum Harmala  (seeds) | No | No | Yes | No |  | Part of feed |
| M. Gallisepticum | | | | | | | | |
| Stipkovits et  al (2004) | Biromedicin Co.Ltd., Budapest,  Hungary | Wheat germ | No | No | Briefly | 2,6-dimethoxi-pbenzoquinone  0.4 mg/g | Not specified | Part of feed |
| Coccidiosis | | | | | | | | |
| Christaki et  al (2004) | Apa-CT, s.r.l.  Italy | Apacox:  Agrimonia eupatoria,  Echinacea angustifolia,  Ribes nigrum,  Cinchona succirubra | No | No | No | No |  | Part of feed |
| Dragan et al  (2010) | No | 1&2. Artemisia annua  (powder & oil)  3. Pimpinella anisum  (oil)  4. Combination (oils) | No | No | No | No |  | Oil mixed in  water  Powder in feed |
| Giannenas  et al (2003) | Meriden Animal  Health Ltd. (Luton,  UK | Orego-Stim:  Origanum vulgare  subsp. hirtum | No | No | No | No |  | Part of feed |
| Matthews et  al (2000) | Finnsugar Bioproducts  Inc.,  Schaumburg, IL  60173--5008. | Betafin-BCR:  Betaine | No | No | No | No |  | Part of feed |
| Oviedo-  Rondón et al  (2006) | Akzo Nobel  Surface Chemistry  LLC, Chicago,  IL | Crina Poultry/  Crina Alternate:  Main compounds:  Thymus  vulgaris, Syzygium  aromaticum, Cinnamonum  zeylanicum,  Curcuma zanthorrhiza,  Piper nigrum | No | No | No | Thymol  Eugenol  Curcumin  Piperin  (No concentrations  given) |  | Part of feed |
| Dragan et al  (2014) | 1. Winnenden  Germany.  2. Fares®  1929 (Romania) | 1. Artemisia Annua:  Leaf powder,  2. A. annua & Foeniculum  vulgare:  Essential oils | Yes | Yes | 1.Yes  2. A. annua:  Yes  F. vulgare:  No | 1.A. annua: Artemisinin,  dihydroartemisinic  acid, artemisinic acid  2.F. vulgare: trans-  Anethole, fenchone,  methyl chavicol, limonene,  O-pinene and  traces of camphene,  sabinene, S-pinene,  myrcene, camphor,  carvone,  anisaldehyde and others  A annua essential oil:  not specified | 1. Highperformance  liquid  chromatography  with photodiode  array detection  (HPLC-UV)  2. F. vulgare: Not  specified | 1.In feed  2.In water |
| Hassan et al  (2008) | No | Gyamopsis  tetragonoloba | No | No | No | No |  | Part of feed |
| Lee et al  (2008) | National Rural  Resources Development  Institute  (Suwon, South  Korea). | Prunus Salicina | No | No | Briefly | No |  | Part of feed |
| Allen (2003) | Triarco Industries,  Patterson,  N.J. | Echinacea purpurea | No | No | No | No |  | Part of feed |
| McDougald  (2014) | Muscadine  Products Corporation  Wray, GA | Muscadine pomace | No | No | Briefly | No |  | Part of feed |
| (Cacho et  al., 2010) | Dafra Pharma  N.V./S.A  Belgium | Artemisia Annua | No | No | No | No |  | Part of feed |
| Necrotic enteritis | | | | | | | | |
| McReynolds  et al., (2009) | Biomin, San  Antonio, TX | PEP125:  Essential oil from  citrus, oregano, annase.  Unspecified plant  extracts, Fructooligosacharides | No | No | No | No |  | Part of feed |
| Engberg et  al., (2012) | Seeds from:  Mediplant, Switzerland | Artemisia annua  1. Dried leaves  2. Extract | Yes | Yes | Yes | 1. Artemisin  2. Essential oil components | 1. Ultra-highperformance  liquid  chromatography  (HPLC)  2. Gas chromatography  (GC)-mass  spectrometry | On top of feed |
| Mitsch et al.,  (2004) | Crina SA, Gland,  Switzerland | 1. Crina Poultry:  Thymus vulgaris,  Syzygium aromaticum,  Cinnamomum  zeylanicum,  Curcuma, zanthorrhiza,  Piper nigrum  2. Product name not  specified. Content as  above and Origanum  vulgare | No | No | No | Essential oil components  1. thymol, eugenol,  curcumin, and piperin  2. thymol, carvacrol,  eugenol, curcumin, and  piperin | Established by gas  chromatography  (but result or analysis not included in  paper) | In feed |
| Ordonez,  Llopis, &  Penalver,  (2008) | Lidervet, S. L.,  Tarragona, Spain | Liderfeed:  Eugenia caryophyllata  (Syzygium aromaticum)  Essential oil | Yes | No | No | 20 % Eugenol | Gas chromatography-  quadrupole  mass spectroscopy | ? |
| Cattle | | | | | | | | |
| Endometritis | | | | | | | | |
| Heuwieser,  Tenhagen,  Tischer,  Lühr, &  Blum, 2000 | PlantaVet | Eucacomp:  Eucalyptus | No | No | No | No |  | Intrauterine  application |
| Mastitis | | | | | | | | |
| Hu et al.  (2001) | Indena SPA  Milan  Italy | Panax Ginseng | Batch no. | No | No | Ginsenoside Rg1 15 % | Not specified | Parenteral |
| Ruminal acidosis | | | | | | | | |
| Balcell et al  (2012) | Exquim S.A.,  Barcelona, Spain | Bioflavex:  Citrus aurantium  Citrus paradisi | No | No | No | Naringine | Not specified | In feed |
| Benchaar et  al. (2012) | Phodé S.A., Albi,  France | ? | No | No | No | Eugenol  Active ingredient purity  >0,99 | Not specified | In feed |
| Devant et al.  (2007) | Phytosynthese,  France | Biostar®:  Cynarin, Siberian  ginseng, fenugreek | No | No | No | No | Not specified | In feed |
| Calf diarrhea | | | | | | | | |
| Oliveira et al.  (2010) |  | Pomegranate seed  extract | No | No | No | 16, 9% Gallic acid  equivalent | Not specified | In feed |
| Bampidis et  al. (2010) | Ecopharm Hellas  SA, Kilkis,  Greece | Oregano  Dried leaves | No | No | Yes | Essential oil yield with  content: carvacrol  854,9 g/kg  thymol 37,8 g/kg  y-terpinene 20,6 g/kg  p-cymene 26,2 g/kg | Not specified | Oral application |
| Pneumonia | | | | | | | | |
| Bednarek et  al. (2002) | Andean  Medicine  Centre BCM,  London | Vilcacora® -  Uncaria tomentosa | No | No | No | No |  | Oral decoction |
| Swine | | | | | | | | |
| E. coli | | | | | | | | |
| Jugl-Chizzola et  al. (2005) | L. Wiberg, Salzburg,  Austria | Thymus vulgaris  Essential oil | Yes (by compounds) | No | No | Essential oil yield 1,66  %  39 % p-cymene 32 %  thymol  3,4 % carvacrol  1,6 % 1,8-cineol  4 % linalool | Gas chromatograhpy  Mass spectroscpy | Oral application  3 days before  weaning, then  part of feed |
| Liu et al.  (2013) | Pancosma SA,  Geneva, Switzerland | Capsicum oleoresin,  Garlic,  Turmeric oleoresin | No | No | No | Capsicum and Turmeric:  6% capsaicin and dihydrocapsaicin  98% curcuminoides  Garlic  40% propyl thiosulfonates | Not specified | Part of feed |
| Manzanilla  et al. (2004) | No | Oregano, Cinnamon  and Mexican pepper | No | No | No | 5% carvacrol, 3% cinnamaldehyde,  and 2%  capsicum oleoresin | Not specified | Part of feed |
| Sads &  Bilkei (2003) | Pecs, Hungary | Oregpig:  Origanum vulgare  (Dried herb combined  with essential oil) | No | No | No | Essential oil: carvacrol  60 g and thymol 55  g/kg | Not specified | Part of feed |
| Salmonellosis | | | | | | | | |
| Turner, Dritz,  Higgins,  Herkelman,  & Minton,  2002 | Turner, Dritz,  Higgins,  Herkelman,  & Minton,  2002 | Quillaja saponaria | No | No | No | No | No | Part of feed |
| Turner, Dritz,  Higgins, &  Minton, (2002) | Acadian Seaplants  Limited | Ascophyllum  nodosum | No | No | No | No |  | Part of feed |
| Janczyk et  al. (2008) | No | "natural identical free  thymol" | No | No | No | Thymol | Not specified | Part of feed |
| van Parys et  al. (2009) | Sanluc  International  nv/Global  Nutrition sa,  Oosterzele,  Belgium | Globatan:  Castanea sativa | No | No | No | No |  | Part of feed |
| PRRS | | | | | | | | |
| Liu, Che, et  al., (2013) | Pancosma SA,  Geneva, Switzerland | Capsicum oleoresin  Garlic  Turmeric oleoresin | No | No | No | Capsicum and Turmeric:  6% capsaicin and dihydrocapsaicin  98% curcuminoides  Garlic  40% propyl thiosulfonates | Not specified | Part of feed |
| Greiner,  Stahly, &  Stabel, (2001) | Wiley Organics,  Coshocton, OH | Soy bean | No | No | No | Genistein  88.8% pure extract | high-performance  liquid chromatography | Part of feed |
| Hermann et  al. (2003) | Nature’s Cathedral,  Inc., Blairstown,  IA | Echinacea purpurea  Ground root | No | Some (harvest  age  and time) | Yes | 0.39% caftaric acid,  0.01% chlorogenic  acid, less than 0.01%  echinacoside, and  1.35% cichoric acid | High-pressure liquid  chromatography | Part of feed |
| Post weaning diarrhea | | | | | | | | |
| Schoene et  al. (2006) | Centre of Renewable  Resources  Dornburg  Thuringia | 1. Foeniculi aetheroleum  (fennel seed)  Essential oil  2. Carvi aetheroleum  (carraway seed)  Essential oil | No | Some | Yes (both) | Anethol, fenchon,  a +b-pinen  etc. in g/kg | capillary gas chromatography | Part of feed |
| Endometritis |  |  |  |  |  |  |  |  |
| Kis & Bilkei,  (2003) | Feed Additives,  Pecs, Hungary | Oregpig:  Origanum vulgare  subsp. hirtum | No | No | No | carvacrol 60 g and  thymol 55 g/kg | Not specified | Not specified |
| Pig nodular worm | | | | | | | | |
| Magi et al.  (2005) | 1-3.Not specified  4. Dansk Droge  A/S | 1. Curbita pepo  (pumpkin)  2. Tancaetum vulgare  (tansy)  3. Acorus calamus  (sweet flag)  4. Allium sativum  (garlic) | No | No | Yes | No | Not specified | Per os |
